# Influence of Low-Level Red Laser Irradiation on the Proliferation, Viability, and Differentiation of Human Embryonic Stem Cell-Derived Mesenchymal Stem Cells

**DOI:** 10.3390/life15071125

**Published:** 2025-07-17

**Authors:** Khalid M. AlGhamdi, Ashok Kumar, Musaad Alfayez, Amer Mahmood

**Affiliations:** 1 Vitiligo Research Chair, Department of Dermatology, College of Medicine, King Saud University, Riyadh 11472, Saudi Arabia; aknirankari@gmail.com; 2 Department of Dermatology, College of Medicine, King Saud University, Riyadh 11472, Saudi Arabia; 3 Stem Cell Unit, Department of Anatomy, College of Medicine, King Saud University, Riyadh 11472, Saudi Arabia; alfayez@ksu.edu.sa (M.A.); amer_dk@yahoo.com (A.M.)

**Keywords:** low-level laser therapy, red laser, cell culture, proliferation, viability, migration, differentiation, tissue engineering, regenerative medicine

## Abstract

The present investigation was conducted to observe the effects of different energy densities of a low-level red laser (LLRL) on human embryonic stem cell-derived mesenchymal stem cells (hESC-MSCs). hESC-MSCs were cultured and irradiated with a LLRL from 0.5 to 5.0 J/cm^2^ at a wavelength of 635 nm. Biological parameters such as proliferation, viability, and migration were observed after 72 h of LLRL irradiation. Compared with the control, LLRL irradiation significantly increased the proliferation and viability of hESC-MSCs from 0.5 to 2.5 J/cm^2^ (*p* < 0.001, *p* < 0.05). LLRL irradiation from 0.5 to 3.0 J/cm^2^ significantly increased the migration of hESC-MSCs (*p* < 0.01). These results revealed that LLRL irradiation at lower energy densities significantly increased the proliferation, viability, and migration of hESC-MSCs. However, higher energy densities were ineffective; this was also true when we examined osteogenic differentiation, as low energy densities of LLRL had a positive effect on differentiation, whereas higher energy densities had a negative effect on alkaline phosphatase activity, Alizarin Red staining and gene expression analysis. In addition, not all stem cell markers were affected by the laser, and a slight decrease in the expression of CD146, which is a stemness marker, was detected, indicating improved differentiation. These findings indicate that low energy densities of LLRL irradiation have positive effects on the proliferation, migration, and differentiation of hESC-MSCs. However, higher energy densities showed inhibitory effects.

## 1. Introduction

The application of mesenchymal stem cells (MSCs) in regenerative medicine is rapidly expanding due to their ability to differentiate into multiple lineages and modulate immune responses. Despite their therapeutic potential, clinical translation is hindered by challenges such as slow proliferation, limited viability during expansion, and inefficient osteogenic differentiation [1,2,3]. Tissue engineering strategies aim to overcome these limitations by enhancing MSC function without compromising cell health.

Low-level laser therapy (LLLT) has emerged as a promising non-invasive approach to modulate cellular behavior. Prior studies have demonstrated that LLLT can enhance MSC proliferation, migration, and differentiation, particularly when parameters like wavelength, energy density, and irradiation duration are optimized [4,5,6,7,8]. Red light lasers (630–680 nm), in particular, fall within the bioactive spectrum and have been shown to stimulate osteogenic markers such as ALP (alkaline phosphatase), Runx2 (runt-related transcription factor 2), and BMP2 (bone morphogenic protein) [9,10,11,12,13,14].

However, despite growing interest, the literature remains inconclusive and inconsistent, especially regarding the optimal energy density and specific effects on human embryonic stem cell-derived MSCs (hESC-MSCs), a highly promising yet underexplored cell source for bone regeneration. Most prior studies focus on adult MSCs (e.g., from bone marrow or adipose tissue), leaving a gap in understanding whether hESC-MSCs respond similarly to red laser stimulation. Additionally, the influence of varying energy densities on both proliferation and differentiation has not been critically compared in a single model system, limiting our ability to define effective therapeutic parameters.

Therefore, this study aimed to evaluate the influence of different energy densities of red laser LLLT on hESC-MSCs, focusing on proliferation, viability, migration, and osteogenic differentiation at both the phenotypic and gene expression levels.

## 2. Materials and Methods

### 2.1. Human Embryonic Stem Cell-Derived Mesenchymal Stem Cell Culture

The Stem Cell Unit, College of Medicine, King Saud University, Riyadh, provided hESC-MSCs for this study [15]. hESC-MSCs were cultured in high-glucose DMEM (Gibco, Thermo Fisher Scientific, Waltham, MA, USA) supplemented with 10% fetal calf serum (FBS; Gibco, Thermo Fisher Scientific, Waltham, MA, USA), 1% essential amino acids (Gibco, Thermo Fisher Scientific, Waltham, MA, USA) and 1% antibiotic-anti-mycotic (Gibco, Thermo Fisher Scientific, Waltham, MA, USA). hESC-MSCs were cultured at 37 °C in a humidified chamber with 5% CO_2_. For subculture, hESC-MSCs were treated with 0.25% trypsin in 0.01% ethylenediamine tetraacetic acid (Gibco, Thermo Fisher Scientific, Waltham, MA, USA) and incubated at 37 °C for 3–5 min. Full medium was then added to stop the reaction. hESC-MSCs were collected by centrifugation at 500× *g* for 5 min, resuspended in complete medium and plated in 48-well plates. Then, the hESC-MSCs were allowed to incubate overnight before red laser treatment. 

### 2.2. Low-Level Red Laser Irradiation

Different energy densities of a low-level red laser (LLRL) at a wavelength of 635 nm were used to irradiate hESC-MSCs as previously described [16,17,18,19,20]. The applied power density was 25 mW/cm^2^ in continuous wave mode during this study. The energy densities of the LLRL were 0.5, 1.0, 1.5, 2.0, 2.5, 3.0, 3.5, 4.0, 4.5, and 5.0 J/cm^2^, and the irradiation duration was 20 to 200 s to irradiate hESC-MSCs along with the control groups (nonirradiated hESC-MSCs).

We irradiated hESC-MSCs only once. The medium of the hESC-MSCs was removed, and sterile phosphate-buffered saline (PBS; Gibco, Thermo Fisher Scientific, Waltham, MA, USA) was added to each well of the hESC-MSCs before irradiation to minimize the loss of laser energy. The laser beam was divergent and had a 1 mm diameter. Irradiation area was 10 mm, which is almost equal to surface of each well in the 48-well plate as explained in our publication [19]. The nonirradiated (control) and irradiated groups were maintained under the same laboratory conditions, such as temperature, humidity, and light, to avoid the influence of second-order variables. The hESC-MSCs were irradiated at room temperature and procedure was performed in a dark room, with the laser as the only light source. Sterile cell culture conditions were maintained during the irradiation procedure, and fresh hESC-MSC growth-promoting medium was added to each well after irradiation for further incubation. The selected energy densities (0.5–5.0 J/cm^2^) were based on our previous study reporting enhanced proliferation, viability and migration of melanocytes without inducing cytotoxic effects at lower doses, on the other hand higher doses showed inhibitory effects [19]. Lower doses have been associated with stimulatory effects on cell proliferation. This range was therefore chosen to capture both proliferative and differentiation responses while avoiding inhibitory or damaging thresholds. During this study, all the experiments were performed in triplicate for statistical analysis.

### 2.3. Cell Proliferation Determination

The proliferation of hESC-MSCs was measured via a 3-[4,5-dimethylthiazol-2-yl]-2,5-diphenyltetrazolium bromide (MTT, Sigma-Aldrich, St. Louis, MO, USA) assay to observe the effect of LLRL. Briefly, hESC-MSCs were plated at a density of 4 × 10^4^ cells per well into 48-well plates (Corning, New York, NY, USA) and cultured overnight at 37 °C. Then, the hESC-MSCs were irradiated with LLRL as described above and further incubated for 72 h. After incubation, the medium was replaced with MTT solution, and the mixture was incubated for 2 h at 37 °C. Then, the MTT mixture was exchanged with isopropanol, shaken for 2 h, and the absorbance at 549 nm was read with a microplate reader (BioTek, Winooski, VT, USA) [21].

### 2.4. Assessment of the Cell Viability Rate

The trypan blue (Sigma-Aldrich, St. Louis, MO, USA) exclusion method was used to assess the viability of hESC-MSCs by counting the number of living cells. Trypan blue dye stains only dead cells; thus, unstained cells are viable. The number of hESC-MSCs was counted, and 4 × 10^4^ cells were seeded into each well of 48-well plates and incubated overnight. The next day, hESC-MSCs were irradiated and further incubated for 72 h. After the experiments, the hESC-MSCs were trypsinized, and the hESC-MSCs were collected in medium. hESC-MSCs were mixed with trypan blue solution (Sigma-Aldrich, St. Louis, MO, USA) and loaded into a hemocytometer counting chamber to quantify the number of living (unstained) cells and the number of dead (stained) cells. The number of LLRL-treated cells was counted and compared with that of control cells to plot graphs of cell viability [19].

### 2.5. Cell Migration Assay

The migration of hESC-MSCs assessed via a scratch assay to observe the effects of LLRL [19]. hESC-MSCs were counted, and cells were seeded into 48-well plates at a density of 4 × 10^4^ cells per well and incubated overnight. A “scratch” was then created in the middle of each well of the 48-well plate with an autoclaved 200 µL pipette tip, after which the hESC-MSCs were irradiated. After irradiation, the PBS, was changed to fresh medium to remove debris, and the hESC-MSCs were further incubated for 72 h. Photographs were captured at 72 h by a camera attached to an inverted phase-contrast microscope (Olympus Corporation, Tokyo, Japan) at a power of 10x. Photographs were captured from three separate “scratch areas” at the same magnification for statistical analysis.

Photographs of the control and irradiated groups were compared, and the number of migrated hESC-MSCs in the “scratch area” was counted to generate graphs for the migration assay. This assay was performed in triplicate. We selected two energy densities from significant and one energy density from non-significant value to show proliferative and inhibitory effects of red laser on migration at lower and higher energy densities.

### 2.6. Osteoblast Differentiation (OB) Analysis

Human mesenchymal stem cells (hMSCs) overexpressing the telomerase reverse transcriptase gene (hTERT) were used as a model for primary human MSCs, which presented similar cellular and molecular traits. The hMSCs used in this study were cultured in DMEM (Gibco-Invitrogen) supplemented with 10% FBS, D-glucose, L-glutamine, sodium pyruvate, penicillin-streptomycin, and nonessential amino acids (all from Gibco, Thermo Fisher Scientific, Waltham, MA, USA). The cells were grown to 70–80% confluence at 50,000 cells/mL. For osteoblast induction, the medium was replaced with differentiation medium containing 10% FBS, penicillin-streptomycin, L-ascorbic acid (Gibco, Thermo Fisher Scientific, Waltham, MA, USA), β-glycerophosphate (Sigma-Aldrich, St. Louis, MO, USA), calcitriol, and dexamethasone (Sigma-Aldrich, St. Louis, MO, USA). Differentiation was carried out in 48-well plates for 10 days used for FACS & genetic analysis and for 21 days used for ALP staining, and the medium was changed three times per week.

### 2.7. Quantitative Alkaline Phosphatase Activity

ALP (alkaline phosphatase) activity assay kit (Abcam, Cambridge, UK) was measured with the BioVision ALP Activity Colorimetric Assay Kit. The cells cultured in 48-well plates were rinsed with PBS before 50 µL of pNPP solution was added and incubated in the dark for 2 min at room temperature. The optical density was measured at 405 nm. We selected three energy densities from significant groups to show proliferative effects of red laser by ALP staining.

Alizarin Red Staining:

After 21 days of culture, cells were washed with PBS and fixed in 4% paraformaldehyde for 15 min at room temperature. Fixed cells were stained with Alizarin Red S (ARS, 40 mM, pH 4.2) for 30 min to assess calcium deposition. Excess dye was removed by washing with distilled water. Stained wells were imaged to visualize mineralization.

Image Analysis:

The intensity of Alizarin Red staining was visually compared between groups. Increased red staining indicates enhanced calcium deposition, suggesting successful osteoblast differentiation.

### 2.8. Flow Cytometry Analysis of Treated and Untreated Cells

Flow cytometry was performed on single cells from the treated and control groups. After LLRL treatment, the cells were harvested, neutralized in serum, and resuspended in FACS buffer at approximately 1,000,000 cells/mL. Staining was conducted via the use of MSC-specific antibodies (CD14APC, CD29PE, CD31FITC, CD34FITC, CD44PE, CD73PE, and CD146PE) and isotype controls (BD Biosciences, San Jose, CA, USA). After 30 min of staining on ice, the samples were washed, and at least 10,000 events per sample were acquired via a FACSCalibur (BD Biosciences, San Jose, CA, USA) and analyzed with Cellquestpro^®^ software version 1.2 (BD Biosciences, San Jose, CA, USA). Flow cytometry analysis for all plots were performed, however due to space we did not include all plots in the manuscript (for all plots see Supplementary Figure S1).

### 2.9. Quantitative Real-Time PCR (qRT-PCR) Procedure

The innuPREP RNA Mini Kit (Analytik Jena, Jena, Germany) was used to extract total RNA, which was quantified with a Nanodrop spectrophotometer (Thermo Fisher Scientific, Waltham, MA, USA). A high-capacity cDNA reverse transcription kit (Thermo Fisher Scientific, Waltham, MA, USA) was used to perform cDNA synthesis. Relative mRNA levels were determined via RT-PCR with a Fast SYBR Green PCR kit (Thermo Fisher Scientific, Waltham, MA, USA) via the comparative CT method and were normalized to the GAPDH reference gene.

### 2.10. Statistical Analysis

Descriptive statistics were analyzed using IBM software SPSS 28.0 version. For inferential statistical analysis, one-way ANOVA was used to compare the mean values of the quantitative variables across the categorical variables, followed by a *post hoc* Tukey HSD test via GraphPad Prism 4.0 (GraphPad Software, San Diego, CA, USA). A *p* value of <0.05 was considered statistically significant.

## 3. Results

### 3.1. Impact of Low-Level Red Laser Treatment on the Proliferation of hESC-MSCs

**Figure 1:**
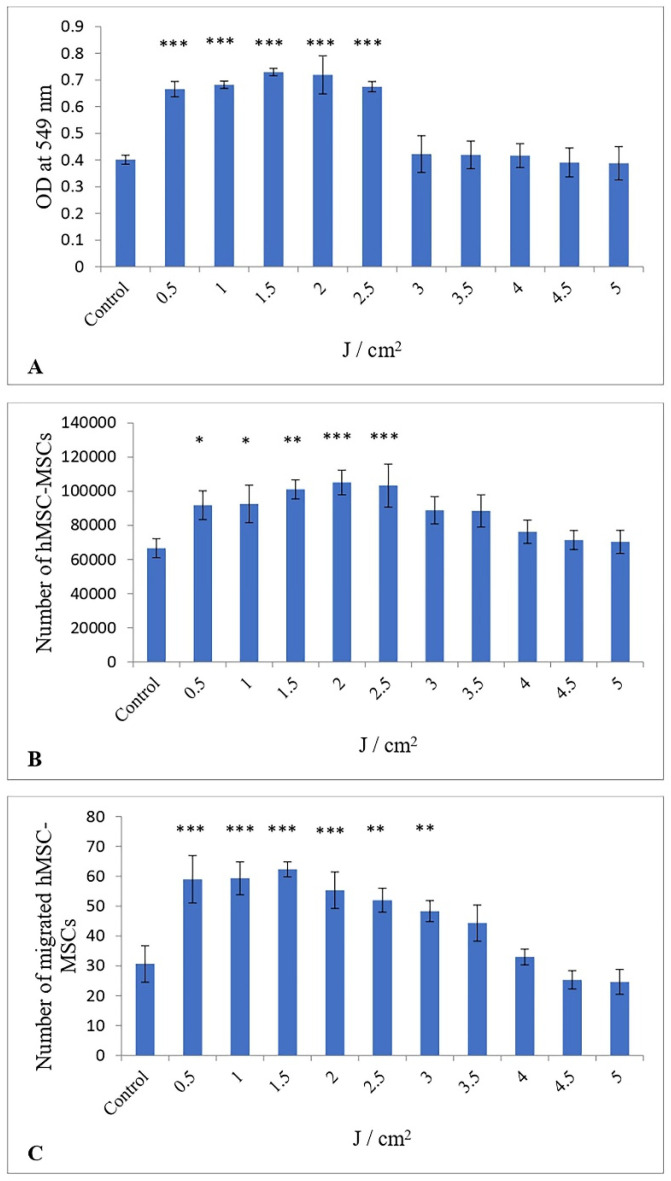
Effects of a low-level red laser from 0.5 to 5.0 J/cm^2^ on the proliferation (**A**), viability (**B**) and migration (**C**) of human embryonic stem cell-derived mesenchymal stem cells (hESC-MSCs). Cultured hESC-MSCs were seeded in 48-well plates, and next day, the cells were exposed to a low-level red laser (635 nm) while in PBS, then further incubated for 72 h. Dimethylthiazol tetrazolium bromide (MTT assay) was used to study proliferation and the number of living hESC-MSCs was counted under a microscope for viability at end of experiment. For migration assay photographs were captured at 72 h, the number of migrated hESC-MSCs in the scratch area was counted, and a graph was generated as described in the Materials and Methods. OD: optical density. The control and treatment groups were compared and the data were analyzed by ANOVA. *p*-values were: * *p* < 0.05; ** *p* < 0.01; *** *p* < 0.001.

The proliferation of hESC-MSCs was assessed via the MTT assay. LLRL significantly increased the proliferation of hESC-MSCs exposed to different energy densities. Figure 1A shows that the proliferation of hESC-MSCs increased by 1.65, 1.69, 1.81, 1.79, 1.68, 1.05, 1.04, 1.03, 0.97, and 0.96-fold at doses of 0.5, 1.0, 1.5, 2.0, 2.5, 3.0, 3.5, 4.0, 4.5, and 5.0 J/cm^2^ energy density, respectively, compared with that of the control, with a maximal and significant effect obtained following 1.5 J/cm^2^ (*p* < 0.001). In addition, LLRL increased proliferation from 3.0 to 4.0 J/cm^2^, but this increase was not significant. On the other hand, LLRL did not increase the proliferation of hESC-MSCs when hESC-MSCs were treated with energy densities ranging from 4.5 to 5.0 J/cm^2^. One-way ANOVA revealed that the effect of LLRL with energy density ranging from 0.5 to 2.5 J/cm^2^ was significantly different from that of the control, and the lowest *p* value was <0.001.

### 3.2. Impact of Low-Level Red Laser Treatment on the Viability of hESC-MSCs

The number of viable cells counted at 72 h following irradiation is shown in Figure 1B. LLRL significantly increased the viability of hESC-MSCs by 1.37, 1.38, 1.51, 1.57, 1.54, 1.33, 1.32, 1.41, 1.07, and 1.05-fold at doses of 0.5, 1.0, 1.5, 2.0, 2.5, 3.0, 3.5, 4.0, 4.5, and 5.0 J/cm^2^, respectively, compared with the control, with a maximal and significant effect obtained following 2.0 J/cm^2^ (*p* < 0.001). One-way ANOVA revealed that LLRL was able to increase the number of viable hESC-MSCs from 3.0 to 5.0 J/cm^2^ compared with the control, but this increase was not statistically significant.

### 3.3. Influence of a Low-Level Red Laser on the Migration of hESC-MSCs

**Figure 2:**
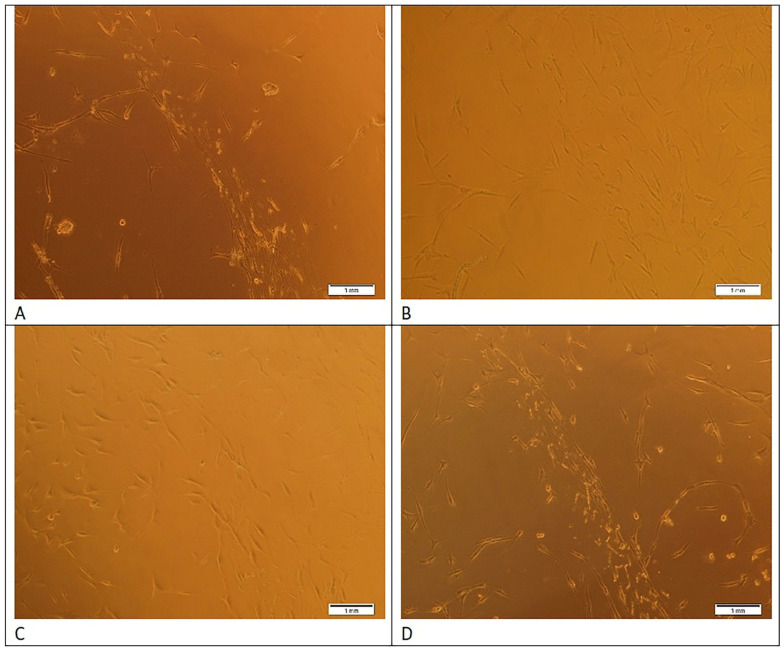
Human embryonic stem cell-derived mesenchymal stem cells were plated in 48-well plate and irradiated with a low-level red laser at energy densities 0.5, 1.0, 4.0 J/cm^2^ and control for the migration assay as explained in material and methods section. Then, cells were further incubated for 72 h. Images of Human embryonic stem cell-derived mesenchymal stem cells were captured using phase-contrast microscope at 72 h after treatment. The control (non-irradiation (**A**)) and treatment groups viz. (**B**) (0.5 J/cm^2^), (**C**) (1.0 J/cm^2^) and (**D**) (4.0 J/cm^2^) were compared and the data were analyzed by ANOVA.

Compared with the control, LLRL significantly increased the number of migrated hESC-MSCs from 0.5 to 3.0 J/cm^2^. As shown in Figure 1C, the number of migrated hESC-MSCs treated with LLRL increased by 1.92, 1.93, 2.03, 1.8, 1.69, 1.57, 1.44, 1.07, 0.82, and 0.8 at doses of 0.5, 1.0, 1.5, 2.0, 2.5, 3.0, 3.5, 4.0, 4.5, and 5.0 J/cm^2^ energy density, respectively, compared with the control, with a maximal and significant effect obtained following 1.5 J/cm^2^ (*p* < 0.001). One-way ANOVA revealed that LLRL significantly increased the number of migrated hESC-MSCs from 0.5 to 3.0 J/cm^2^ compared with the control, and the lowest *p* value was <0.01. However, LLRL at 3.5 and 4.0 J/cm^2^ increased the number of migrated cells, but the difference was not significant. On the other hand, LLRL failed to increase the migration of hESC-MSCs at energy densities of 4.5 and 5.0 J/cm^2^ relative to that of the untreated group. Photomicrographs of hESC-MSCs treated with LLRL revealed that migration was most effective at a 1.5 J/cm^2^ energy density compared to control, and the migration of hESC-MSCs was significantly greater (Figure 2).

The descriptive statistics for the triplicate samples comparing the control group and the ten experimental groups are presented in Table 1. The dataset exhibits a normal distribution, increasing from the lowest dose of 0.5 to 2, followed by a declining curve from the 2.5 dose. The peak mean cell proliferation occurs at a dose of 1.5, with a mean optical density of 0.73 ± 0.01 (95% CI 0.72–0.74). Given that Cohen’s d effect size is 16.1, which exceeds the 0.8 threshold established by Cohen’s criteria, the impact of the experiment is considered substantially large. Similarly, the peak viability of cells reached at a dose of 2, achieving a mean 105,184 ± 5902.9 (95% CI 98,504.9–111,864.2) and a larger effect size of 4.3 indicating a significant experimental effect. The maximum viability of cells was attained at a dosage of 2, with a mean value of 105,184 ± 5902.9 (95% CI 98,504.9–111,864.2) and a substantial effect size of 4.3, indicating a significant experimental impact implying larger practical applications. Furthermore, the migration of cells reached the peak at 1.5 dosage attaining a mean value of 62.3 ± 2 (95% CI 60–64.5). The sample attained an effect size of 4.8 signifying a large experimental effect compared to the control group. The variance generally showed closer observations for proliferation and migration of cells, but viability showed a scattering of data from the mean for control as well as all the ten experimental groups indicating a higher degree of variability.

### 3.4. Low-Level Red Laser Stimulation Enhances the Osteoblast Differentiation of hESC-MSCs

**Figure 3:**
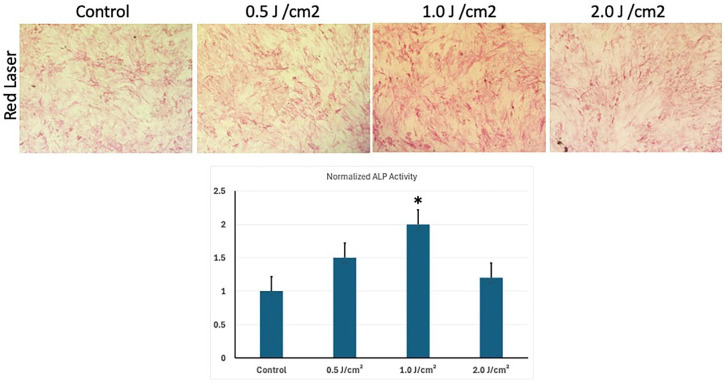
Alkaline Phosphatase (ALP) staining of Human embryonic stem cell-derived mesenchymal stem cells (hESC-MSCs) stimulated with a low-level red laser and then differentiated into osteoblasts. hESC-MSCs were plated in 48-well plate and irradiated with 0.5, 1.0, 4.0 J/cm^2^ as explained in material and methods section. Control: Baseline ALP activity showing normal differentiation levels without laser treatment. 0.5 J/cm^2^: Minimal effect on differentiation, similar to the control. 1.0 J/cm^2^: Noticeable increase in ALP activity, suggesting enhanced differentiation at this intermediate laser intensity. 2.0 J/cm^2^: Decreased ALP activity compared with that at 1.0 J/cm^2^ (20X images). Lower panel: Quantification of ALP activity in cells treated with red laser at varying energy densities (0.5 J/cm^2^, 1.0 J/cm^2^, and 2.0 J/cm^2^) compared to the untreated control. ALP activity was normalized to the control, showing a peak at 1.0 J/cm^2^ and a slight decrease at 2.0 J/cm^2^, indicating dose-dependent effects. * *p* < 0.05.

ALP activity was measured to observe the ability of LLRL-treated hESC-MSCs to differentiate into osteoblasts. Three different energy densities of LLRL, 0.5, 1.0, and 2.0 J/cm^2^, were applied to hESC-MSCs, with control and osteoblast-induced media used to observe the differentiation of hESC-MSCs toward the osteoblast lineage (Figure 3). (Selection of energy densities 0.5, 1.0, and 2.0 J/cm^2^ was based on MTT (Proliferative assay) results. These energy densities showed best effect with significant increment in proliferation of cells as explained in Figure 1). After LLRL irradiation, the hESC-MSCs were incubated for 10 days, after which differentiation was measured by ALP activity quantitative and qualitative. These results revealed that LLRL gradually increased differentiation (Figure 3). These results demonstrate that while LLRL treatment can influence osteoblast differentiation, its effectiveness is dependent on the energy density, with an optimal effect observed at 1.0 J/cm^2^ (Figure 3 bar diagram). Higher energy densities appear to diminish the differentiation potential, highlighting the need for careful optimization of laser parameters to increase osteogenic differentiation (Figure 3).

**Figure 4:**
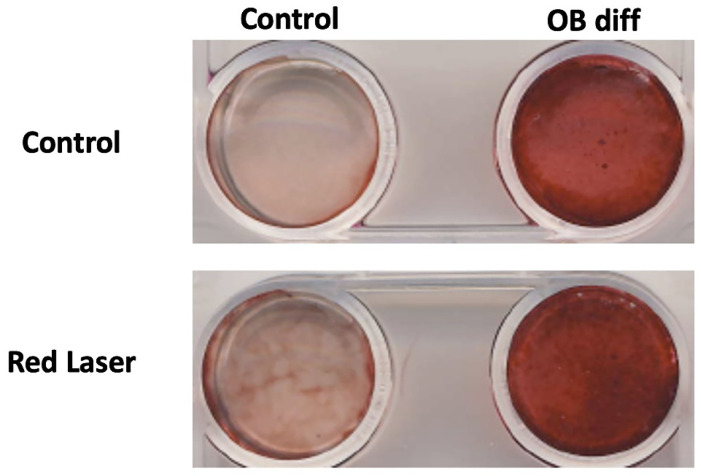
Effect of Red Laser on Osteoblast Differentiation Assessed by Alizarin Red Staining. Representative images of Alizarin Red staining in mesenchymal stem cells (MSCs) cultured under control conditions (top row) or exposed to red laser treatment (bottom row). The left column represents the unstimulated control group, while the right column represents cells cultured in osteogenic differentiation (OB diff) media. Increased mineral deposition, indicated by darker red staining, is observed in the osteogenic differentiation groups, with enhanced staining in the red laser-treated 1.0 J/cm^2^, condition compared to the untreated control. This suggests that red laser exposure may promote osteoblast differentiation and matrix mineralization.

Following alkaline phosphatase (ALP) staining, Alizarin Red staining was performed to assess extracellular matrix mineralization, a key indicator of osteoblast differentiation. The results demonstrated a significant increase in calcium deposition in the osteogenic differentiation (OB diff) groups, as indicated by the intense red staining (Figure 4).

In the control group (non-differentiated MSCs), minimal staining was observed, suggesting low baseline mineralization. In contrast, the OB diff group exhibited strong Alizarin Red staining, confirming successful osteogenic differentiation. Notably, cells treated with Red Laser (1.0 J/cm^2^) showed an enhanced staining intensity compared to their non-irradiated counterparts, suggesting that laser treatment may promote mineralization and accelerate osteoblast differentiation (Figure 4).

These findings align with the ALP staining results, further supporting the hypothesis that red laser irradiation enhances osteogenic differentiation by stimulating both early-stage (ALP activity) and late-stage (mineral deposition) markers of osteoblast development.

### 3.5. Flow Cytometry Analysis

**Figure 5:**
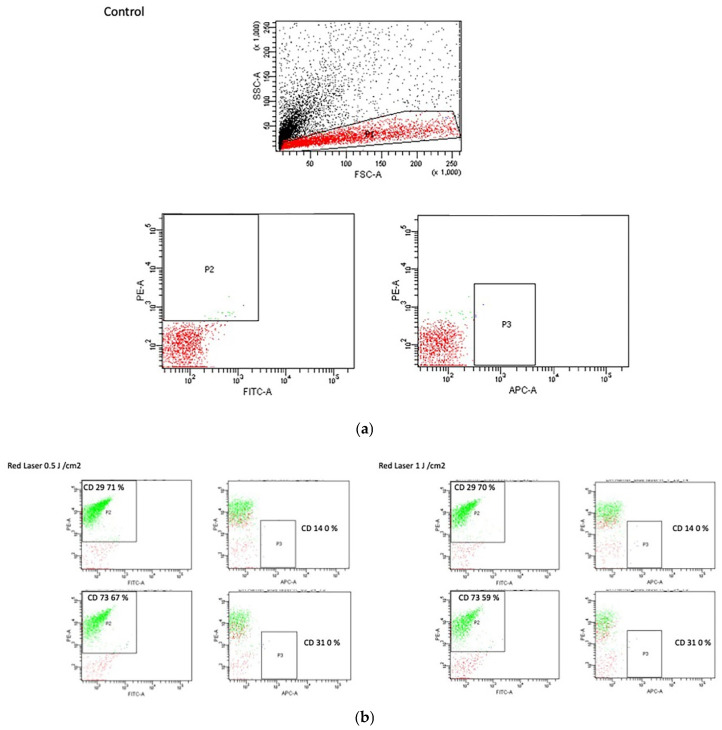
(**a**) FACS analysis of the control sample (without laser treatment), showing forward scatter (FSC) and side scatter (SSC) alongside the isotype control, which was consistent across low-level red laser treatments and various energy densities. **Top panel**: forward scatter (FSC) vs. side scatter (SSC) plot, illustrating the distribution of cell populations in the control sample. **Bottom left panel**: isotype control for FITC-A, depicting the P2 gating region. **Bottom right panel**: isotype control for APC-A, showing the P3 gating region. The isotype control results were the same for low-level red laser treatments across all energy densities. (**b**) Flow cytometry (FACS) analysis results of cells treated with a low-level red laser at doses of 0.5 J/cm^2^ and 1 J/cm^2^. The cells were analyzed for the expression of the CD29, CD73, CD14, and CD31 markers.

The FACS data of hESC-MSCs treated with LLRL at three different energy densities, 0.5, 1.0, and 2.0 J/cm^2^, revealed significant insights into the impact of laser treatment on stem cell marker expression. We observed that higher concentrations of LLRL resulted in a reduction in key stem cell markers. This trend suggests a potential influence of LLRL on the stemness and differentiation potential of hESC-MSCs, with higher laser intensities leading to decreased expression of markers associated with stem cell maintenance and multipotency. Figure 5a shows hESC-MSCs under control conditions without any treatment and with the ISO-type control. We observed that there was no background staining with any fluorescence.

Figure 5b shows the results of FACS analysis of hESC-derived mesenchymal stem cells (hESC-MSCs) treated with LLRL at energy densities of 0.5 J/cm^2^ and 1 J/cm^2^, assessing the expression of the surface markers CD29, CD73, CD14, and CD31. The expression of CD29 was slightly lower in LLRL-treated cells than in control cells. At 0.5 J/cm^2^, CD29 expression was 29.71%, whereas at 1 J/cm^2^, it was 29.70%, indicating minimal variation with increasing laser intensity. A similar trend was observed for CD73 expression. In cells treated with 0.5 J/cm^2^, CD73 expression was 73.67%, and for the 1 J/cm^2^ treatment, it was 73.59%. These findings suggest that LLRL treatment slightly decreased CD73 expression compared with that in control cells. The expression levels of CD14 and CD31 remained relatively stable across both treatment conditions. CD14 expression was consistently 14.0% for both the 0.5 J/cm^2^ and the 1 J/cm^2^ treatments. CD31 expression was 31.0% for both treatment intensities, indicating that LLRL treatment had no significant effect on these markers. These results indicate that LLRL treatment at the tested energy densities led to a slight reduction in the expression of CD29 and CD73 in the MSCs, with no significant changes observed in the CD14 and CD31 expression levels.

Table 2 presents the FACS analysis results for hESC-MSCs under control conditions, highlighting the expression levels of various general markers. The data revealed a high expression level of CD29, with 81.70% of the total cells expressing this marker. CD29, also known as integrin β1, is critical for cell adhesion and interaction with the extracellular matrix, indicating the robust presence of adhesion capabilities in control hESC-MSCs. No expression (0%) was detected for CD14, CD34, or CD31. CD14 is a monocyte/macrophage marker, CD34 is a hematopoietic stem cell marker, and CD31 is an endothelial cell marker. The absence of these markers confirmed that the control hESC-MSCs did not spontaneously differentiate into these lineages under standard culture conditions. A significant proportion of cells (75.40%) express CD73, an ecto-5′-nucleotidase involved in maintaining stem cell multipotency and differentiation. This high expression level suggests that the control hESC-MSCs retain a high degree of stemness and multipotent characteristics. CD146 was expressed in 55.80% of the total cells. CD146, or melanoma cell adhesion molecule, is associated with vascular smooth muscle cells and pericytes, indicating that a subset of the hESC-MSC population may be predisposed toward these lineages. A smaller percentage (5.30%) of the cells expressed CD44, a cell surface glycoprotein involved in cell–cell interactions, migration, and adhesion. The lower expression level of CD44 compared to other markers suggests a limited role in these functions under control conditions. Overall, the control data confirmed the expected expression profiles of general markers in hESC-MSCs, which maintained high levels of stemness markers (CD29 and CD73) while showing no spontaneous differentiation toward hematopoietic or endothelial lineages.

Table 3 summarizes the effects of different intensities of LLRL treatment (0.5 J/cm^2^, 1.0 J/cm^2^, and 2.0 J/cm^2^) on the expression levels of various surface markers (CD29, CD14, CD73, CD34, CD146, CD31, and CD44) in mesenchymal stem cells (MSCs).

The expression of CD29 decreases progressively with increasing laser intensity, from 71% at 0.5 J/cm^2^ to 69.70% at 1.0 J/cm^2^, and further decreases to 65% at 2.0 J/cm^2^. No expression (0%) of CD14 or CD34 was detected across all laser intensities. The expression of CD73 also tends to decrease with increasing laser intensity, decreasing from 67.20% at 0.5 J/cm^2^ to 59.10% at 1.0 J/cm^2^ and finally to 56.40% at 2.0 J/cm^2^. In contrast, CD146 expression increased with increasing laser intensity, starting at 25.60% at 0.5 J/cm^2^, increasing to 31.40% at 1.0 J/cm^2^, and reaching 34.50% at 2.0 J/cm^2^. No expression (0%) of CD31 was detected across all laser intensities. The expression of CD44 decreases slightly with increasing laser intensity, from 7.50% at 0.5 J/cm^2^ to 5.90% at 1.0 J/cm^2^ and finally to 5% at 2.0 J/cm^2^. Overall, Table 3 highlights the differential effects of LLRL treatment on various MSC surface markers, demonstrating both decreases and increases in marker expression depending on the intensity of the laser treatment.

### 3.6. Gene Expression Analysis of Key Osteoblastic Genes

**Figure 6:**
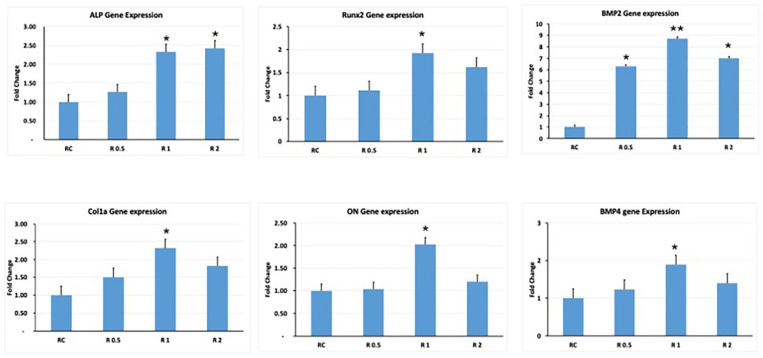
qRT-PCR analysis of gene expression in hESC-MSCs following low-level red laser treatment. The figure presents the fold change in gene expression of various osteogenic markers in hESC-MSCs after low-level red laser treatment at different energy densities (0.5 J/cm^2^, 1 J/cm^2^, and 2 J/cm^2^). The genes analyzed included ALP, Runx2, BMP2, Col1a, ON, and BMP4. The control group is indicated as RC (red laser control). * *p* < 0.05, ** *p* < 0.01.

qRT-PCR analysis revealed the effects of LLRL exposure on the expression of key osteogenic markers in hESC-MSCs. These data indicate that LLRL treatment at various energy densities modulates the expression of these markers, which are crucial for osteoblast differentiation and function (Figure 6).

ALP expression increased significantly at 1.0 J/cm^2^ and 2.0 J/cm^2^, suggesting that LLRL treatment enhances early osteogenic differentiation at these intensities. Runx2 showed a significant increase in expression at 1.0 J/cm^2^, indicating that this intensity optimally promotes the expression of Runx2, a critical transcription factor for osteogenesis. The expression remained elevated at 2.0 J/cm^2^ but was slightly lower than that at 1.0 J/cm^2^. BMP2 had the highest expression at 1.0 J/cm^2^, with a slight decrease at 2.0 J/cm^2^. BMP2 is essential for bone formation, and its increased expression suggests an optimal osteogenic response at 1.0 J/cm^2^. The expression of Col1a significantly increased at 1.0 J/cm^2^ and 2.0 J/cm^2^, indicating enhanced production of collagen type I, a major component of the bone extracellular matrix, at these laser intensities. Osteonectin (ON) expression increased significantly at 1.0 J/cm^2^, suggesting that this laser intensity enhances the expression of osteonectin, a glycoprotein involved in bone mineralization. The expression remained high at 2.0 J/cm^2^ but was slightly lower than that at 1.0 J/cm^2^. Finally, BMP4 presented the highest expression at 1.0 J/cm^2^, with moderate increases at 0.5 J/cm^2^ and 2.0 J/cm^2^. BMP4, like BMP2, plays a crucial role in bone development, indicating a strong osteogenic response at 1.0 J/cm^2^ (Figure 6).

## 4. Discussion

Many investigators have reported that LLLT has become the main tool for enhancing the proliferation of various cell types [22], such as keratinocytes [23], fibroblasts [24], and osteoblasts [25], as well as many other stem cells [26,27,28]. LLLT is also known as photomodulation, which can enhance or inhibit biological parameters depending on the application of wavelengths and energy densities. In general, red (600–700 nm) and near-infrared (NIR, 780–1100 nm) wavelengths are used during the photomodulation process [29].

The aim of the present investigation was to enhance biological parameters, including the proliferation and differentiation of hESC-MSCs, via LLRL irradiation. In this study, different energy densities of LLRL at a wavelength of 635 nm were applied to hESC-MSCs, and their proliferation, viability, migration, and differentiation after LLRL treatment were examined.

In this study, energy densities of LLRL ranging from 0.5 to 5.0 J/cm^2^ were applied to observe the effects on the proliferation of hESC-MSCs at lower doses and the inhibitory effects at high doses. LLLT has been reported to reduce the metabolism of cells and damage photoreceptors at high doses, which leads to cell death [4]. Proliferation was measured via the MTT assay. During the MTT assay, the optical densities of the treated and nontreated (control) groups were measured after irradiation and compared with those of the control groups. These results showed that LLRL significantly increased proliferation at low energy densities (0.5 to 2.5 J/cm^2^). On the other hand, high energy densities inhibited the growth of hESC-MSCs. Similarly, in a previously published study, red light increased proliferation at 3.0 J/cm^2^ [30] and LLLT significantly promote proliferation of bone marrow mesenchymal stem cells at 2.0 J/cm^2^ [31], which is similar to our results. These data strengthened our investigation.

Viability, a biological parameter, was measured via trypan blue staining. The unstained viable cells represent living cells, and the stained nonviable cells are dead cells [19]. Our results showed that LLRL at lower energy densities from 0.5 to 2.5 J/cm^2^ significantly increased the viability of hESC-MSCs. Another biological parameter, migration, was measured via a scratch assay to observe the effect of LLRL. It is a simple, effortless, and low-cost method [19]. A scratch or gap area was created with the help of a sterile micropipette in a 48-well plate containing hESC-MSCs. Compared with the control, a greater number of hESC-MSCs were moved to the center to close the gap after LLRL irradiation. These results showed that LLRL from 0.5 to 3.0 J/cm^2^ significantly increased the number of migrated hESC-MSCs to close the gap. Phase-contrast photographs of hESC-MSCs were captured after LLRL irradiation to observe the migration of hESC-MSCs (Figure 2). It was observed that hESC-MSCs treated with low energy densities moved toward the center of the wells, which indicates that cell proliferation occurred.

These results proved that LLRL enhanced the biological parameters of hESC-MSCs, such as proliferation, viability, and migration, at low energy densities (Figure 1 and Figure 2). LLRL at lower energy densities can be useful for stem cell-based therapy.

In terms of osteogenic differentiation, the data indicate that low energy densities positively impact differentiation, as evidenced by enhanced ALP activity, Alizarin Red staining, and the upregulation of osteogenic genes. This observation is consistent with findings by Stein et al. [32], who demonstrated that controlled laser exposure can enhance osteogenic differentiation in stem cells. However, the inhibitory effects observed at higher energy densities further support the notion that overstimulation via high-intensity lasers can disrupt normal differentiation processes, possibly through excessive heat or oxidative stress, which has been noted in previous studies by Pereira et al. [33]. The results from this qPCR analysis suggest that LLRL treatment can effectively enhance the osteogenic differentiation of MSCs, particularly at an energy density of 1.0 J/cm^2^. This energy density appears to be the most beneficial for the upregulation of a wide range of osteogenic markers, including ALP, Runx2, BMP2, Col1a, ON, and BMP4. The observed decline in gene expression at higher energy densities, such as 2.0 J/cm^2^, indicates that there is an optimal threshold for laser treatment, beyond which the benefits may diminish or reverse. The increased expression of these markers strongly indicates enhanced differentiation of hESC-MSCs into osteogenic lineages, reflecting their progression toward osteogenesis.

These findings are significant for regenerative medicine and tissue engineering, particularly because they focus on hESC-MSCs rather than the adult MSCs commonly used in previous LLLT studies [1,2]. By carefully optimizing the laser parameters, it may be possible to maximize the therapeutic benefits while minimizing any adverse effects, thereby improving outcomes in clinical applications involving bone healing and repair.

Interestingly, the expression of stemness markers remained largely unaffected by laser treatment, with the exception of a slight decrease in CD146 expression at higher densities. CD146, which is associated with stemness and endothelial progenitor cells, has been linked to the regulation of differentiation pathways. The decrease in CD146 suggests a potential shift toward a more differentiated state, which aligns with the enhanced osteogenic differentiation observed at lower energy densities. This finding is consistent with the work of earlier reports [34], which discussed the dual role of CD146 in both maintaining stemness and facilitating differentiation under specific conditions.

This study has several limitations that should be acknowledged. First, the experiments were conducted in vitro, which may not fully replicate in vivo conditions, where laser penetration and biological complexity are significantly different. Second, while the study demonstrated the effects of low-level red laser therapy on hESC-MSCs, further research is required to explore the long-term effects and mechanisms underlying the observed phenomena. Third, the limited sample size and focus on specific energy densities may restrict the generalizability of the findings. Lastly, higher energy densities were not extensively explored due to their cytotoxic effects, which limited the scope of understanding the threshold of laser therapy effectiveness.

However, LLLT exerts its effects through the activation of several key molecular pathways that enhance osteogenic differentiation [35]. One primary mechanism is the stimulation of mitochondrial cytochrome c oxidase, leading to increased ATP production and improved cellular energy metabolism. Additionally, LLLT generates controlled levels of reactive oxygen species (ROS), which act as signaling molecules to activate pathways such as MAPK and PI3K/Akt, both of which are critical for cell proliferation and differentiation. The upregulation of osteogenic markers like BMP2 and Runx2 observed in this study aligns with the activation of the TGF-β/BMP signaling pathway. This pathway plays a pivotal role in promoting extracellular matrix deposition and mineralization, facilitating the differentiation of hESC-MSCs into osteoblasts. Furthermore, LLLT-induced ROS may enhance Smad signaling downstream of BMP2, synergizing with Runx2 to regulate osteogenic commitment.

While our study demonstrates that low-level RED laser (LLRL) irradiation enhances proliferation, migration, and osteogenesis in hESC-MSCs in vitro, translating these findings to in vivo settings requires consideration of several biological complexities. Unlike in vitro cultures, tissue penetration, vascularization, and immune responses in living organisms may alter the effects of LLRL. Moreover, alternative wavelengths—such as near-infrared (800–1000 nm)—may penetrate deeper than 635 nm and could induce distinct osteogenic responses, warranting further comparative experiments. Previous research has shown that LLLT can enhance bone healing in animal models, suggesting its potential for clinical applications. Additionally, LLRL may modulate osteogenic signaling pathways such as Wnt/β-catenin and BMP/Smad, which are crucial for bone formation and mesenchymal stem cell differentiation. However, our study was conducted in monolayer cultures, which do not fully replicate the dynamic interactions of cells within the extracellular matrix in vivo. As part of future work, we plan to (1) evaluate additional laser wavelengths for deeper or differential tissue penetration, (2) incorporate Western blot analysis to confirm protein-level upregulation of osteogenic markers, and (3) test our optimized dose in an animal model of bone injury to solidify translational relevance and confirm efficacy. Investigating these areas will help highlight the unique behavior of hESC-MSCs under different laser parameters and confirm the distinction from previous studies focusing on adult MSCs. By acknowledging these limitations and proposing future directions, this study provides a foundation for further research into the therapeutic potential of LLLR in regenerative medicine.

ALP expression and activity serve as well-established indicators of early osteoblast differentiation, and our data provide strong evidence of osteogenic commitment in response to low-level red laser (LLRL) treatment. Additionally, previous studies have demonstrated that ALP activity precedes mineral deposition, making it a reliable marker for evaluating osteogenesis before extracellular matrix mineralization occurs.

Clinically, the findings demonstrate the potential of LLLT as a noninvasive therapy for enhancing bone regeneration. Applications may include accelerating fracture healing, improving the efficacy of bone grafts, and supporting craniofacial and dental surgical outcomes. These results highlight the translational significance of LLLT and reinforce the need for expanded experimental approaches—including comparisons across different wavelengths and thorough in vivo validation—to delineate the precise mechanisms and optimize application protocols.

## 5. Conclusions

This study demonstrates that low energy densities of LLRL (0.5 to 2.5 J/cm^2^) can enhance proliferation, viability, and induce early osteogenic gene expression in hESC-MSCs, suggesting its potential utility in regenerative applications. Flow cytometry showed a reduction in stem cell markers such as CD29 and CD73 at higher energy densities, along with an increase in CD146—a marker sometimes associated with vascular lineage or differentiation. However, changes in surface markers alone are not definitive proof of lineage commitment. Therefore, these findings should be interpreted cautiously.

qRT-PCR results revealed significant upregulation of osteogenic markers (ALP, Runx2, BMP2, Col1a, ON, BMP4) at 1.0 J/cm^2^, indicating activation of osteogenic pathways. These transcriptional changes may suggest stimulation of the BMP2/Runx2/ALP signaling axis. To confirm functional osteogenesis, we plan to validate these results through protein-level analyses (e.g., Western blotting for Runx2, ALP) and mineralization assays (e.g., Alizarin Red S or calcium quantification).

While the 635 nm wavelength was effective in our setup, we acknowledge that different wavelengths may have varying penetration depths and bioactivity. Future work will explore additional red and near-infrared wavelengths to define optimal parameters for laser-enhanced osteogenic differentiation.

These findings suggest that LLRL irradiation, particularly at optimized lower energy densities, holds promise for clinical applications in stem cell-based therapies. The findings suggest that LLLT could be a non-invasive adjunct therapy for superficial bone defects, dental applications, or craniofacial regeneration. Future research should focus on elucidating the underlying molecular mechanisms and optimizing laser parameters for specific therapeutic applications.

## Figures and Tables

**Table 1:** Descriptive statistics for the triplicate samples comparing the control group and the ten experimental groups.

	Control	0.5 J/cm^2^	1.0 J/cm^2^	1.5 J/cm^2^	2.0 J/cm^2^	2.5 J/cm^2^	3.0 J/cm^2^	3.5 J/cm^2^	4.0 J/cm^2^	4.5 J/cm^2^	5.0 J/cm^2^
Proliferation (Optical Density)											
Mean	0.4	0.66	0.68	0.73	0.72	0.67	0.42	0.42	0.41	0.39	0.38
Standard Deviation	0.01	0.02	0.01	0.01	0.06	0.01	0.05	0.04	0.03	0.04	0.05
Confidence Intervals	0.39–0.4	0.64–0.68	0.67–0.69	0.72–0.74	0.65–0.78	0.64–0.68	0.36–0.48	0.37–0.46	0.37–0.46	0.34–0.43	0.32–0.43
Variance	0.0001	0.0005	0.0001	0.0001	0.004	0.0002	0.003	0.001	0.001	0.001	0.002
Cohen’s *d* Effect size	16.1										
**Viability (no. of cells)**											
Mean	66,666	91,851	92,592	101,110	105,184	103,332	88,888	88,517	76,295	71,481	70,369
Standard Deviation	4536	6869.1	8950.2	4536	5902.9	10,304.2	6541.8	7715.8	5543.4	4566.2	5543.1
95% Confidence Intervals	61,533.4–71,799.2	84,078.3–99,624.2	82,464.3–102,720.2	95,977.7–106,243.5	98,504.9–111,864.2	91,672.5–114,992.6	81,485.7–96,290.9	79,786.5–97,248.7	70,022.7–82,568.4	66,313.9–76,648	64,096.5–76,641.5
Variance	20,575,720.2	47,184,910.9	80,107,050.9	20,575,720.2	34,844,910.8	106,176,790.2	42,795,720.2	59,534,293	30,729,766.8	20,850,260.6	30,725,816.2
Cohen’s *d* Effect size	4.3										
**Migration**											
Mean	30.6	59	59.3	62.3	55.3	52	48.3	44.3	33	25.3	24.6
Standard Deviation	4.9	6.4	4.4	2	5	3.2	2.8	4.9	2.2	2.5	3.4
95% Confidence Intervals	25–36.1	51.3–66.7	54.3–64.3	60–64.5	49.6–60.9	48.4–55.6	45.1–51.4	38.7–9.8	30.5–35.5	22.4–28.1	20.7–28.4
Variance	24.8	42	20.2	4.2	24.8	10.6	8.2	24.2	4.6	6.2	11.5
Cohen’s *d* Effect size	4.8										

**Table 2:** FACS analysis of key MSC markers in hESC-MSCs under control conditions.

Control	Percentage of Total Cells
CD29	81.70%
CD14	0%
CD73	75.40%
CD34	0%
CD146	55.80%
CD31	0%
CD44	5.30%

**Table 3:** hESC-MSCs were treated with different energy densities (0.5, 1.0, and 2.0 J/cm^2^) of a low-level red laser and then subjected to FACS analysis.

Red Laser	0.5 J/cm^2^	1.0 J/cm^2^	2.0 J/cm^2^
CD29	71%	69.70%	65%
CD14	0%	0%	0%
CD73	67.20%	59.10%	56.40%
CD34	0%	0%	0%
CD146	25.60%	31.40%	34.50%
CD31	0%	0%	0%
CD44	7.50%	5.90%	5%

## Data Availability

Data is contained within the article or Supplementary Materials.

## Supplementary Materials

The following supporting information can be downloaded at: https://www.mdpi.com/article/10.3390/life15071125/s1, Supplementary Figure S1: Flow cytometry analysis of MSC surface markers after red laser treatment.
