# Supplementary material for: Influence of Low-Level Red Laser Irradiation on the Proliferation, Viability, and Differentiation of Human Embryonic Stem Cell-Derived Mesenchymal Stem Cells
Source: Life (Basel). 2025 Jul 17;15(7):1125. doi: 10.3390/life15071125 (PMC12300213; doi:10.3390/life15071125)

Supplementary Figure S1
Red Laser FACS Plots

Supplementary Figure *S*1. Flow cytometry analysis of MSC surface markers after red laser treatment.

- Flow cytometry plots show the expression of characteristic MSC markers (CD29, CD73) and negative markers (CD14, CD34/CD31) in untreated control cells and cells exposed to red laser at 0.5 J/cm^2 and 1 J/cm^2. Control cells expressed high levels of CD29 (80%) and CD73 (75%) with no detectable CD14 or CD34. Laser-treated cells at 0.5 J/cm^2 maintained MSC identity with CD29 at 71% and CD73 at 67%. At 1 J/cm^2, a slight reduction in CD73 (59%) and CD29 (70%) was observed, while CD14 and CD31 remained absent, indicating no differentiation toward hematopoietic or endothelial lineages. These results suggest laser doses preserve MSC phenotype with minor marker modulation.

Control Cells

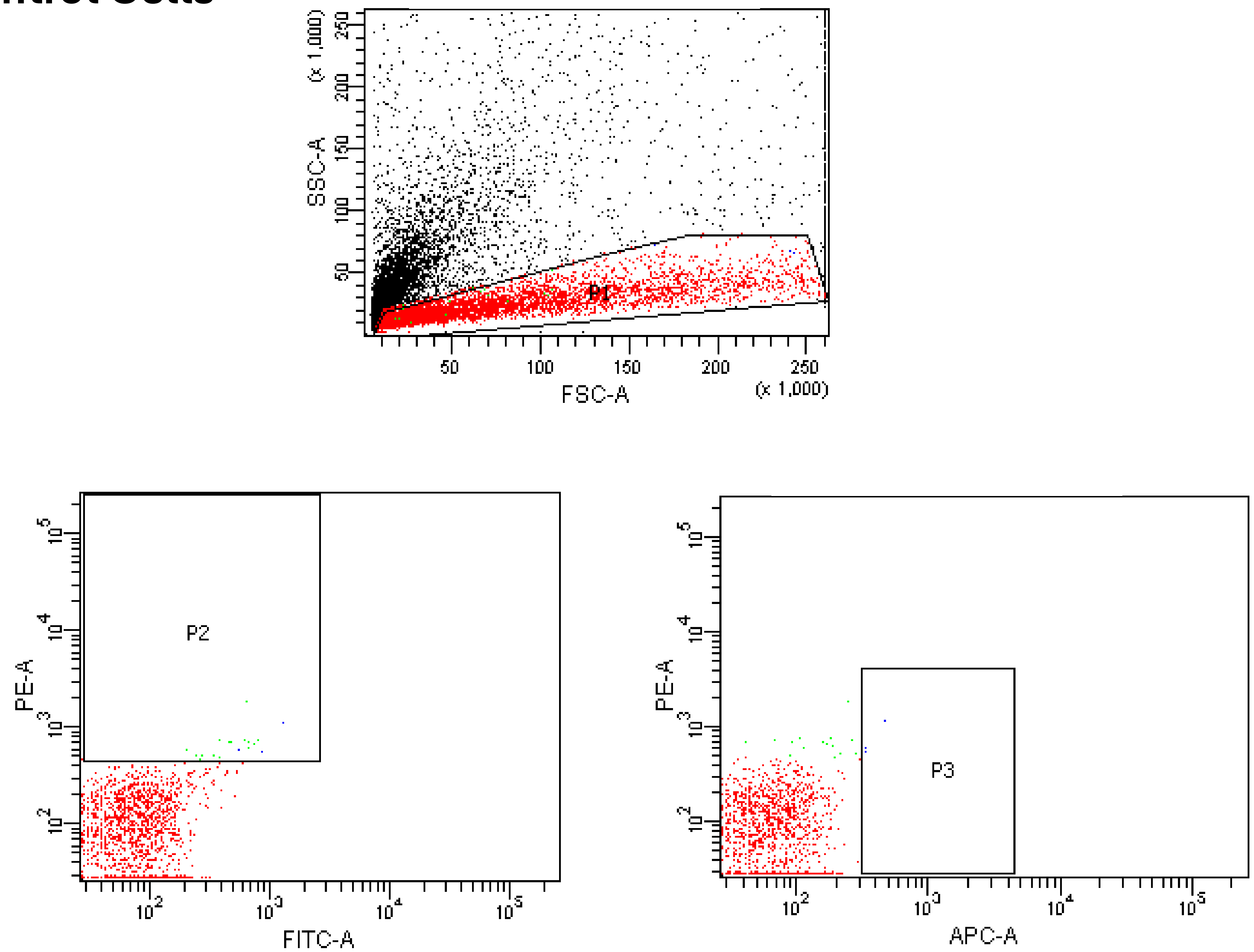

Control

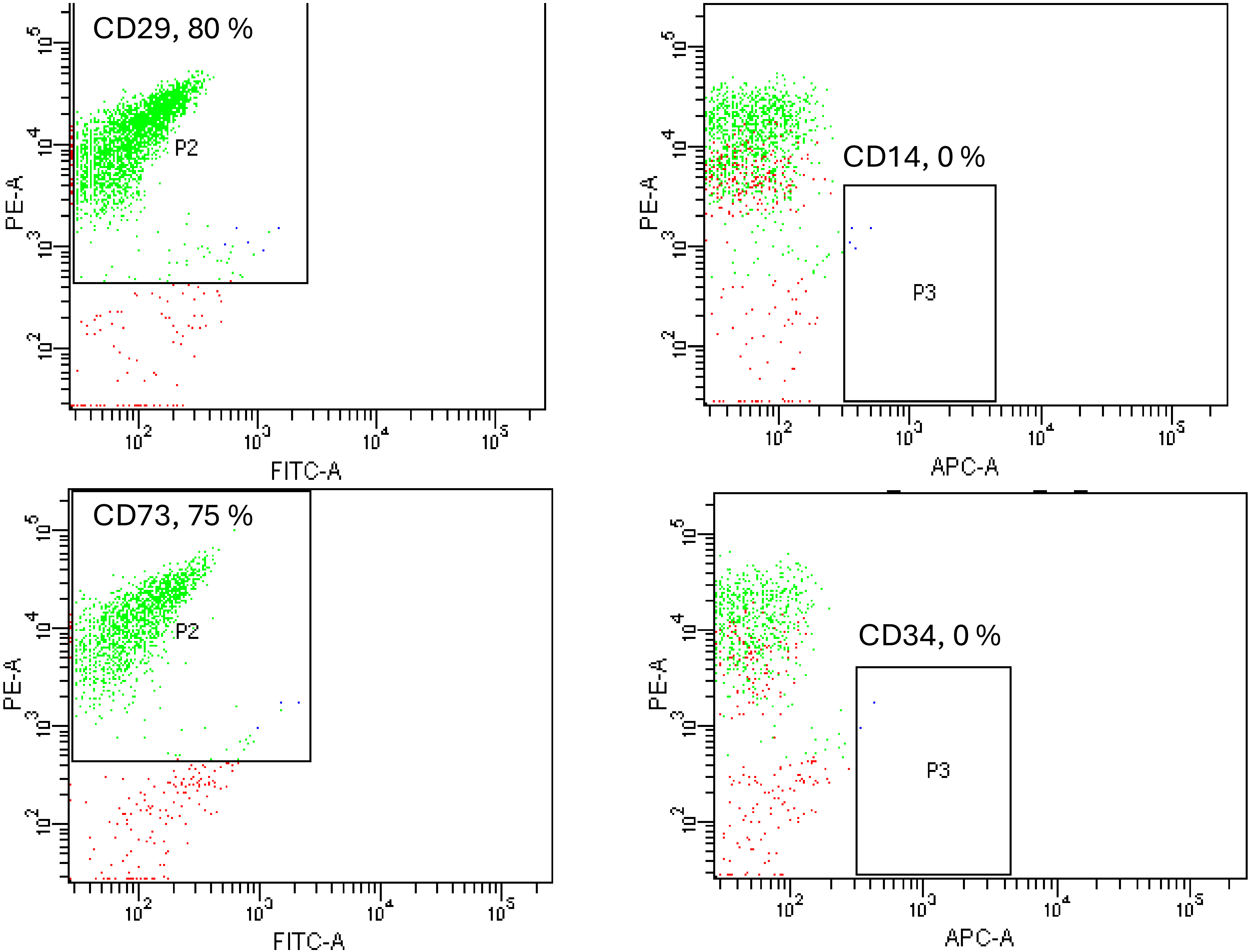

CD29, 80 %
P2
PE-A
FITC-A
CD14, 0 %
P3
PE-A
APC-A
CD73, 75 %
P2
PE-A
FITC-A
CD34, 0 %
P3
PE-A
APC-A

Red Laser 0.5 J /cm2

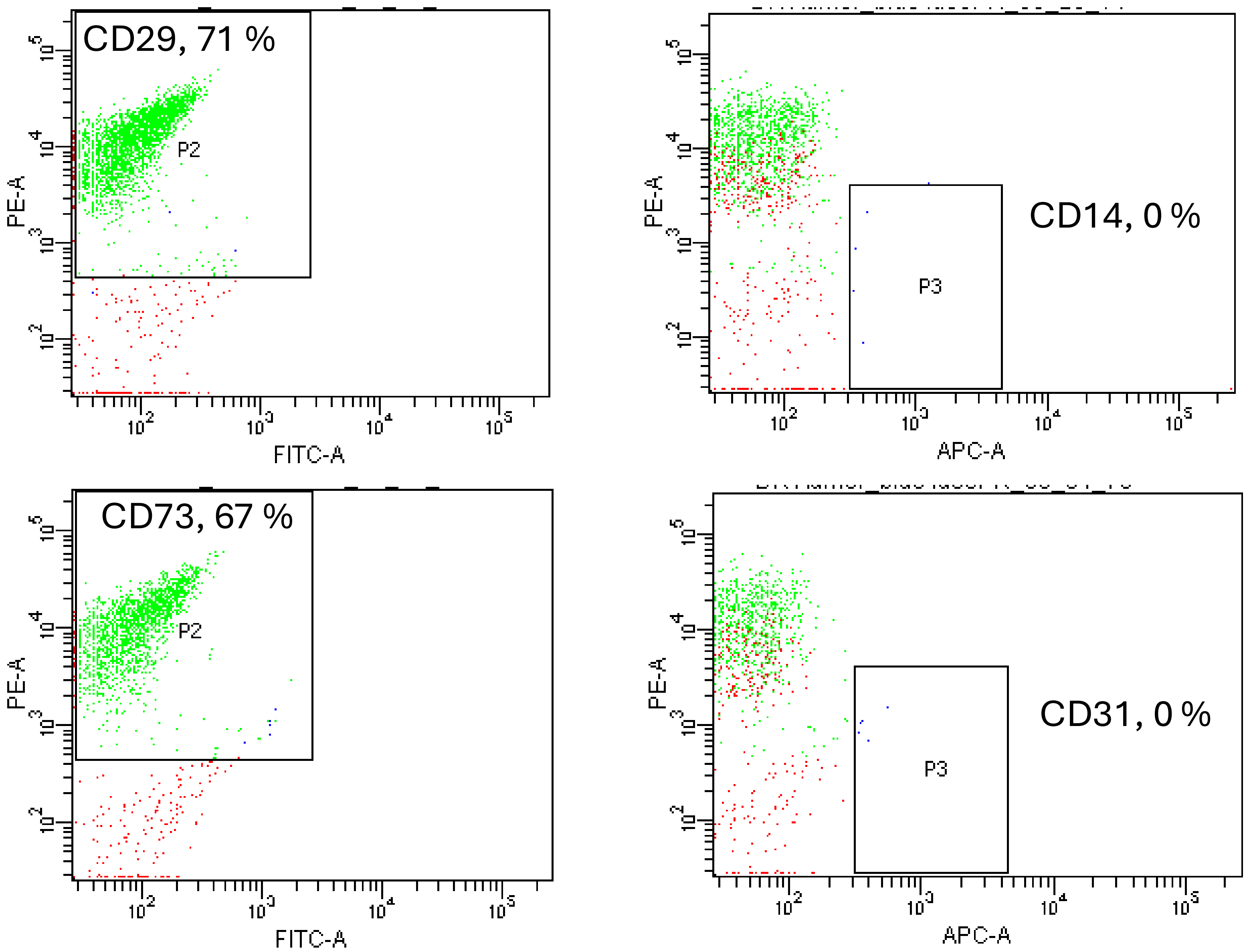

Red Laser 1 J /cm2

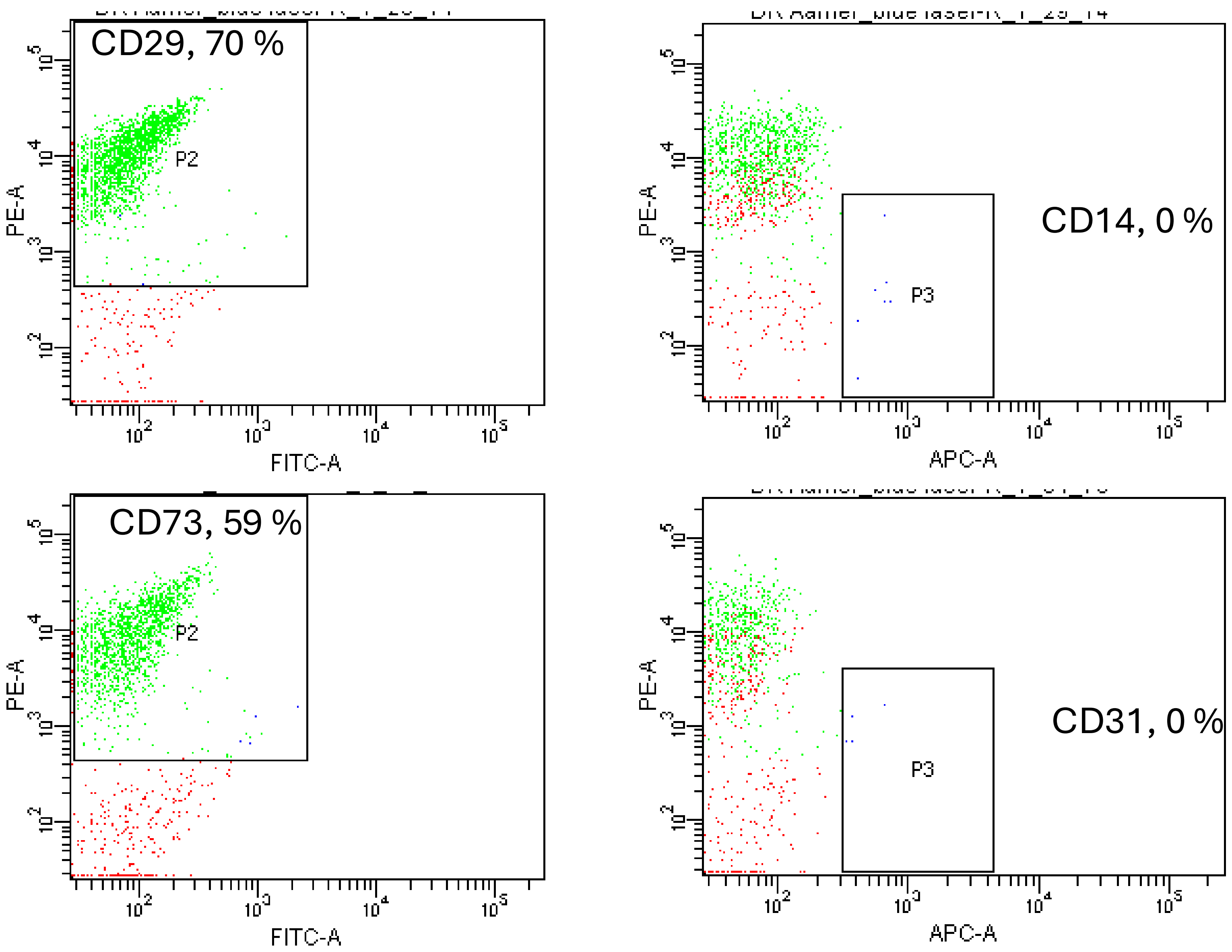

Supplement: Supplementary file 1 [file life-15-01125-s001.zip › life-3720967-supplementary.pdf]
